# Snoring in primary school children and domestic environment: A Perth school based study

**DOI:** 10.1186/1465-9921-5-19

**Published:** 2004-11-04

**Authors:** Guicheng Zhang, Jeffery Spickett, Krassi Rumchev, Andy H Lee, Stephen Stick

**Affiliations:** 1 School of Public Health, Curtin University of Technology, GPO Box U1987, Perth, WA 6845, Australia; 2 Department of Respiratory Medicine, Princess Margaret Hospital for Children, Roberts Road, Subiaco, WA 6008, Australia

## Abstract

**Background:**

The home is the predominant environment for exposure to many environmental irritants such as air pollutants and allergens. Exposure to common indoor irritants including volatile organic compounds, formaldehyde and nitrogen dioxide, may increase the risk of snoring for children. The aim of this study was to investigate domestic environmental factors associated with snoring in children.

**Methods:**

A school-based respiratory survey was administered during March and April of 2002. Nine hundred and ninety six children from four primary schools within the Perth metropolitan area were recruited for the study. A sub-group of 88 children aged 4–6 years were further selected from this sample for domestic air pollutant assessment.

**Results:**

The prevalences of infrequent snoring and habitual snoring in primary school children were 24.9% and 15.2% respectively. Passive smoking was found to be a significant risk factor for habitual snoring (odds ratio (OR) = 1.77; 95% confidence interval (CI): 1.20–2.61), while having pets at home appeared to be protective against habitual snoring (OR = 0.58; 95% CI: 0.37–0.92). Domestic pollutant assessments showed that the prevalence of snoring was significantly associated with exposure to nitrogen dioxide during winter. Relative to the low exposure category (<30 μg/m^3^), the adjusted ORs of snoring by children with medium (30 – 60 μg/m^3^) and high exposures (> 60 μg/m^3^) to NO_2 _were 2.5 (95% CI: 0.7–8.7) and 4.5 (95% CI: 1.4–14.3) respectively. The corresponding linear dose-response trend was also significant (P = 0.011).

**Conclusion:**

Snoring is common in primary school children. Domestic environments may play a significant role in the increased prevalence of snoring. Exposure to nitrogen dioxide in domestic environment is associated with snoring in children.

## Background

Snoring occurs when there is an obstruction to the free flow of air through the airways at the back of the mouth and nose. The prevalence of habitual snoring in children has been reported to vary between 3.2 and 11%. Infrequent snoring is present in 17–27% of all children [1-3]. A study of young Australian children (2–5 years old) found the prevalence of snoring to be 10.5% [4].

Approximately one third of children who snore regularly have obstructive sleep apnea syndrome (OSAS) [5]. A few studies have claimed that snoring in children can affect neurocognitive function, behaviour and blood pressure to some extent even in the absence of apnea [6,7]. Thus, concerns about causes of snoring and prevention strategies for children have arisen among both professional medical workers and parents.

Numerous risk factors for snoring and OSAS have been reported including enlarged adenoids and/or tonsils, obesity, allergies or other causes of nasal obstruction, and exposure to environmental tobacco smoke (ETS) [8-10]. However, there has been very little research on exposure to environmental irritants, other than ETS, as contributing factors for snoring in children. The home is the predominant environment for exposure to many environmental irritants such as allergens and air pollutants. We hypothesized that high levels of exposure to common indoor irritants including volatile organic compounds, formaldehyde and nitrogen dioxide, could increase the risk of snoring. The aim of this study, therefore, was to investigate domestic environmental factors associated with snoring in children.

## Methods

### Study design

Nine hundred and ninety-six (996) school children, aged between 4 and 12 years, were recruited from four primary schools within the Perth metropolitan area. Parents/guardians of the children completed a questionnaire related to respiratory health of their children and domestic environments. A sample of 88 children, aged 4–6 years, was then selected randomly to participate in an indoor air quality assessment of their domestic environments. Ethics approval was obtained from the Human Research Ethics Committee of Curtin University of Technology.

### Respiratory survey

The survey instrument adopted was taken from a questionnaire on respiratory health and indoor air quality [11]. Some questions related to respiratory symptoms and domestic environments have been modified in order to conform to the study objectives. The questionnaire included two parts: the first part covered questions related to children's health and demographic characteristics, the second part consisted of questions about the home environment. Several terms relevant to the study were defined as follows. Children who had asthma were classified as "ever asthma", while those reported having asthma attack or taking any asthma medication within the past 12 months were regarded as "current asthma". Children who had coughed up phlegm on most days over a period of three months were referred to having "chronic productive cough. "habitual snoring" was defined as snoring more than 4 times per week, whereas "infrequent snoring" meant snoring less than 4 times per week. In this context, "snoring" included both "habitual snoring" and "infrequent snoring".

The questionnaires were distributed to parents by school teachers and later collected from the classrooms. The survey was conducted between March and April 2002. A consent form was signed by each participating parent or guardian. The response rate was 62.5%.

### Domestic air pollutant assessment

A sample of 88 year one and pre-primary students was randomly selected from participants of the respiratory survey for domestic air pollutant monitoring. Among them, 34 (38.6%) children were snorers (20 habitual and 14 infrequent). Two home visits were subsequently carried out during the winter of 2002 and summer of 2003 to measure indoor volatile organic compounds (VOCs), formaldehyde and nitrogen dioxide levels.

VOCs were collected in the living room by active sampling using charcoal sorbent tubes. The air-sampling rate was 1 L/min with sampling undertaken for 10 hours during daytime. The analyses were performed using a Perkin Elmer Autosystem XL gas chromatograph equipped with a flame ionization detector. Eleven common compounds were identified and quantified by comparing the retention times: benzene, chlorobenzene, 1,2-dichlorobenzene, 1,3-dichlorobenzene, 1,4-dichlorobenzene, ethylbenzene, styrene, toluene, m-xylene, o-xylene and p-xylene. Their total amount was expressed as total VOCs (TVOCs). Formaldehyde (HCHO) and nitrogen dioxide (NO_2_) were collected by a passive sampling method in both the living room and the child's bedroom for 24 hours. Formaldehyde was analyzed using high-performance liquid chromatography [12].

Nitrogen dioxide was analyzed by a photometric method [13]. The method utilized a Palmes diffusion tube, containing stainless steel screens coated with trithanolamine (TEA), which was used as an absorbent. The concentrations of NO_2 _were measured based on the quantity of the nitrogen dioxide gas transferred through the tube to the absorbent by molecular diffusion during a given exposure period [14,15].

### Data entry and statistics analysis

Preliminary data screening and cleaning were conducted prior to statistical analysis. Associations between the prevalence of snoring and environmental and geographic factors and respiratory symptoms were examined using Chi-square tests. Multivariate logistic regression analysis was undertaken to estimate the risk of snoring adjusting for possible confounders. Since the distributions of VOCs, HCHO and NO_2 _were positively skewed, geometric means (GM) of these variables were calculated after applying a logarithmic transformation. All statistical analyses were performed using the SPSS package Version 10.0.

## Results

Of the 996 participants, 985 children (98.9%) had intact records for snoring. There were 248 children (24.9%; 95% CI: 21.2%–28.6%) reported infrequent snoring and 151 children (15.2%; 95% CI: 12.6%–17.8%) suffered from habitual snoring.

### Snoring by age and gender

Table 1 shows the prevalence of infrequent and habitual snoring by age and gender. Boys had a slightly higher rate of snoring than girls, but the difference was not statistically significant. The rates of habitual snoring decreased significantly with age (P = 0.03).

**Table 1:** Prevalence of infrequent and habitual snoring by age and gender

	Gender						
	Boys		Girls				
	n	%	n		%		P
Infrequent snoring	130	25.9	118		24.4		>0.05
Habitual snoring	82	16.3	69		14.3		>0.05
	Age						
	< 7 years		7 – 9 years		> 9 years		P
	n	%	n	%	n	%	

Infrequent snoring	77	26.6	70	25.5	100	24.0	>0.05
Habitual snoring	58	20.1	42	15.3	51	12.2	0.03

The prevalences of respiratory symptoms, asthma and other allergic conditions were significantly different among non-snoring, infrequent snoring, and habitual snoring children, with habitual snorers having the highest rates. Results are presented in Table 2. A significant association (P < 0.001) was evident between snoring and respiratory symptoms, asthma and other allergic conditions.

**Table 2:** Snoring and respiratory symptoms, asthma and other allergic conditions

	Non-snoring (N = 586)		Infrequent snoring (N = 248)		Habitual snoring (N = 151)		
	n	%	n	%	n	%	P
Phlegm with a cold	145	24.7	97	39.1	66	44.0	<0.001
Phlegm without a cold	38	6.5	31	12.5	25	16.6	<0.001
Chronic productive cough	12	2.1	13	5.2	14	9.3	<0.001
Wheeze during or after exercise	71	12.1	54	21.8	37	24.5	<0.001
Wheeze without exercise	47	8.0	27	10.9	26	17.2	<0.001
Any current wheeze	110	18.8	83	33.5	59	39.3	<0.001
Dry cough at night without a cold	134	22.9	98	39.7	66	43.7	<0.001
Ever asthma	140	23.9	86	34.7	56	37.1	<0.001
Current asthma	83	14.3	62	25.0	36	24.2	<0.001
Allergic rhinitis or hay fever	217	37.6	123	49.6	92	61.7	<0.001

### Snoring and household characteristics

Table 3 shows the proportion rates of various household characteristics. The snoring and non-snoring groups were similar in terms of "gas cooking", "dampness at home" and "carpet in child's bedroom". However, children suffering from infrequent snoring or habitual snoring were more likely to live in "smoking" households (P = 0.004). Children with pets at home seemed to be less likely to develop habitual snoring (P = 0.02).

**Table 3:** Snoring and household characteristics

		Non-snoring		Infrequent snoring		Habitual snoring		
	N	n	%	n	%	n	%	P
Type of cooking								
Gas cooking	565	331	57.5	155	63.0	79	53.0	>0.05
Electric cooking	221	142	24.7	43	17.5	36	24.2	>0.05
Gas and electric cooking	185	103	17.9	48	19.5	34	22.8	>0.05
Dampness at home								
Damp patch	85	49	8.5	25	10.2	11	7.5	>0.05
Condensation	273	153	26.7	74	30.2	46	30.7	>0.05
Mould	167	92	18.1	42	19.2	33	24.1	>0.05
Other characteristics								
Carpet in child's bedroom	827	491	83.9	208	84.2	128	85.3	>0.05
Smoking household	432	235	41.3	113	46.3	84	56.4	0.004
Pet at home	787	474	82.7	203	83.2	110	73.3	0.022

To further investigate the impact of passive smoking and pet ownership on snoring, logistic regression analysis was undertaken, controlling for confounders age, gender, asthma and other allergic conditions. The results indicated that passive smoking increased the risk of habitual snoring significantly (OR = 1.77; 95% CI: 1.20–2.61), while having pets decreased the risk (OR = 0.58; 95% CI: 0.37–0.92). However, the corresponding effects of passive smoking and pet ownership on infrequent snoring were statistically not significant. The Hosmer-Lemeshow statistic confirmed adequacy of the fitted logistic regression model (P > 0.10).

### Snoring and indoor pollutant exposure

The levels of pollutants exposure were similar between houses of habitual snorers and houses of infrequent snorers. To facilitate analysis, data from the two groups were combined to improve statistical power for comparison with houses of the non-snoring children. Table 4 shows the pollutant measurements. The levels of TVOCs and HCHO were not significantly different between houses of snorers and non-snorers regardless of season. However, the geometric means of NO_2 _concentration in the living rooms of snoring children were higher. In particular, the levels of NO_2 _in snoring children's bedroom were significantly higher than those in non-snoring children's bedroom during winter.

**Table 4:** Snoring and indoor pollutants

		Houses of non-snoring children				Houses of snoring children				
		n^1^	GM^2^	Min	Max	n^1^	GM^2^	Min	Max	P
TVOCs										
Living room	Summer	47	11	1	254	32	15	2	204	>0.05
	Winter	52	15	1	247	34	22	1	575	>0.05
HCHO										
Living room	Summer	48	7	ND	34	32	6	ND	26	>0.05
	Winter	51	15	2	92	33	19	ND	92	>0.05
Bedroom	Summer	48	9	ND	126	32	8	ND	47	>0.05
	Winter	49	16	2	84	33	18	2	98	>0.05
NO_2_										
Living room	Summer	48	37	11	244	32	41	8	511	>0.05
	Winter	51	38	9	314	32	48	6	345	>0.05
Bedroom	Summer	48	32	6	293	32	31	6	199	>0.05
	Winter	50	33	6	267	32	56	8	511	0.015

ND = not detectable

^1 ^Missing data or lost to follow-up present

^2 ^Geometric mean of pollutant concentration (μg/m^3^)

Recognizing that the main source of indoor NO_2 _could be a gas heater and/or gas cooker, we compared NO_2 _concentration between houses with and without a gas heater in the child's bedroom. The results confirmed that NO_2 _levels (GM: 49 μg/m^3^, 95% CI: 37–65 μg/m^3^) in houses with a gas heater were significantly higher than those (GM: 27 μg/m^3^, 95% CI: 20–38 μg/m^3^) recorded in houses without a gas heater.

Logistic regression analysis was next conducted to assess the dose-response relationship between bedroom exposure to NO_2 _during winter and snoring in children.

Based on the empirical NO_2 _distribution, the monitored households were classified as: 'low' exposure (<30 μg/m^3^), 'medium' exposure (30 – 60 μg/m^3^) and 'high' exposure (> 60 μg/m^3^). After adjusting for age, gender, asthma, passive smoking and pet ownership, domestic NO_2 _exposure level was still positively associated with snoring, the ORs being 2.5 (95% CI: 0.7–8.7) for medium exposure and 4.5 (95% CI: 1.4–14.3) for high exposure. There was also evidence of a linear dose-response relationship (P = 0.011 for trend).

## Discussion

Snoring is an important symptom and major risk factor for obstructive sleep apnea [5]. Several studies in Italy and Thailand reported that the prevalence of habitual snoring varied from 4.9 to 34.5% in primary school children [5,16,17], while the prevalence of snoring was 10.5% according to a study of Australian children aged 2–5 years [4]. The present study found the prevalence of habitual snoring among primary school children in Perth was 15.2%, and 24.9% of the children had infrequent snoring. The total prevalence of snoring was 40.1%. That the participants had high rates of current asthma (18.7%) and allergy (44.0%) (allergic rhinitis or hay fever) may explain the apparently high snoring prevalence taking into account the link between snoring and asthma and allergy.

The prevalence of snoring among older children was significantly lower than that of younger children. No significant difference in snoring prevalence between boys and girls was observed, which appeared to be consistent with the literature [16,18].

Strong associations were also found between snoring and respiratory symptoms, asthma and other allergic conditions, as in previous studies [10,19,20].

In relation to the domestic environment, passive smoking was identified as a major risk factor for habitual snoring and consistent with other studies [10,21]. An interesting finding was the observed inverse relationship between snoring and pet ownership. There is evidence in the literature suggesting that pet ownership in early life can protect against the development of allergic disease [22]. Although the protective effect of pet ownership on habitual snoring was significant after controlling for allergic diseases, the mechanism that led to a lower risk of snoring remains to be investigated.

Unlike TVOCs and HCHO, it appears that domestic exposure to NO_2 _was significantly associated with snoring. It should be remarked that the low exposure threshold was set below the annual value of 40 μg/m^3 ^recommended by WHO [23], whereas the high exposure cut off is higher than the guideline value. Our results suggested that high exposure to NO_2_could increase the risk of snoring by 4.5 times. A previous study reported that children aged 5–12 years had a 20% increased risk of respiratory symptoms and disease for each increase of 28.3 μg/m^3 ^in NO_2_concentrations (2-week average), when the weekly average concentrations were in the range 15–128 μg/m^3 ^or possibly higher [23]. Another study in Australia confirmed the link between NO_2 _exposure from gas appliances and the prevalence of respiratory symptoms [24]. Our results also suggested that exposure to NO_2 _was related to gas heating during winter.

Although the effect of NO_2 _exposure on snoring was significant even after adjustment for asthma, atopy and other confounding factors, caution must be taken when interpreting the NO_2 _findings and further investigation is required before they can be generalized to the pediatric population at large. A limitation of this cross-sectional study is that only 9% of the study sample was monitored for environmental testing due to budget and other constraints. Nevertheless, this subgroup of children did not differ significantly from the whole sample or other populations of young children in Perth [25,26] with respect to home environment, respiratory symptoms and atopy. Secondly, the causal effects of NO_2 _could not be determined because the measurements of exposure and illness were taken at the same time. The assessment of snoring was retrospective in relation to the time of environmental monitoring. Moreover, the significant association between snoring and NO_2 _exposure in winter may be attributed to NO_2 _emission from gas heaters in conjunction with low ventilation during the winter season.

As for potential mechanisms for this association, there is little in the literature that can directly explain how exposure to NO_2 _might result in snoring. Although there is evidence to suggest that exposure to NO_2 _is associated with development of allergic disease [27], the observed association between NO_2 _exposure and snoring is independent of atopy. Snoring occurs due to upper airway obstruction during sleep. The obstruction commonly occurs at the level of the nasal turbinates as with anterior rhinitis or the nasopharynx due to adenoid hypertrophy. Exposure of airway epithelium *in vitro *results in the release of inflammatory cytokines and adhesion molecules [28]. Therefore, it is possible that exposure to NO_2 _increases upper airway inflammation, resulting in mucosal oedema and airway obstruction. Alternatively, upregulation of ICAM1 the primary ligand for rhinovirus [28] could increase the susceptibility to, or severity of upper respiratory tract infection, resulting in upper airway oedema and/or adenoid hypertrophy. Finally, it has been suggested that NO_2 _increases lipid membrane fluidity [29] that in turn can alter receptor-ligand interactions. Thus NO_2 _exposure might produce changes in cell-cell and cell-pathogen interactions that could result in altered upper airway physiology. Given the high prevalence of snoring in our population and the knowledge that snoring is a significant risk factor for obstructive sleep apnea, the mechanisms that might underpin the association between NO_2 _exposure and snoring require further study.

In conclusion, the present study shows that snoring is common among primary school children in Perth, and snoring is associated with other respiratory symptoms. Passive smoking increases the risk of snoring in children but pet ownership may decrease the risk. The level of nitrogen dioxide in domestic environment is positively associated with the prevalence of snoring in children.

## Authors' contributions

GZ, JS, KR, SS Study design, coordination and management

GZ, JS, KR Field measurement and laboratory work

AHL, GZ Data analysis and interpretation of results

GZ, JS, KR, AHL, SS Preparation and revision of the manuscript
